# Potential therapies targeting nuclear metabolic regulation in cancer

**DOI:** 10.1002/mco2.421

**Published:** 2023-11-29

**Authors:** Yanjie Chen, Jie Xu, Xiaoyi Liu, Linlin Guo, Ping Yi, Chunming Cheng

**Affiliations:** ^1^ Department of Obstetrics and Gynecology The Third Affiliated Hospital of Chongqing Medical University Chongqing China; ^2^ Department of Microbiology and Immunology The Indiana University School of Medicine Indianapolis Indiana USA; ^3^ Department of Radiation Oncology James Comprehensive Cancer Center and College of Medicine at The Ohio State University Columbus Ohio USA

**Keywords:** cancer therapy, DNA methylation, histone modifications, nuclear metabolic enzymes, nuclear metabolic signaling, tumor metabolism

## Abstract

The interplay between genetic alterations and metabolic dysregulation is increasingly recognized as a pivotal axis in cancer pathogenesis. Both elements are mutually reinforcing, thereby expediting the ontogeny and progression of malignant neoplasms. Intriguingly, recent findings have highlighted the translocation of metabolites and metabolic enzymes from the cytoplasm into the nuclear compartment, where they appear to be intimately associated with tumor cell proliferation. Despite these advancements, significant gaps persist in our understanding of their specific roles within the nuclear milieu, their modulatory effects on gene transcription and cellular proliferation, and the intricacies of their coordination with the genomic landscape. In this comprehensive review, we endeavor to elucidate the regulatory landscape of metabolic signaling within the nuclear domain, namely nuclear metabolic signaling involving metabolites and metabolic enzymes. We explore the roles and molecular mechanisms through which metabolic flux and enzymatic activity impact critical nuclear processes, including epigenetic modulation, DNA damage repair, and gene expression regulation. In conclusion, we underscore the paramount significance of nuclear metabolic signaling in cancer biology and enumerate potential therapeutic targets, associated pharmacological interventions, and implications for clinical applications. Importantly, these emergent findings not only augment our conceptual understanding of tumoral metabolism but also herald the potential for innovative therapeutic paradigms targeting the metabolism–genome transcriptional axis.

## Graphical abstract

1. Cell metabolism and gene control are significantly integrated and regulated by one another.

2. There is a positive feedback loop of metabolism‐epigenetics‐gene regulation which control cancer progression.

3. The discovery of metabolic enzymes and their functions in the nucleus will provide more insights into cancer therapy.

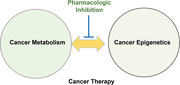

## 1 INTRODUCTION

The reciprocal modulation of genetic information and metabolic dysregulation serves as a cornerstone in cancer pathogenesis. The crosstalk between these two domains is an imperative subject that holds significant implications for both our understanding of cancer progression and therapeutic innovation. In eukaryotic cells, genetic information is primarily housed within chromatin structures, whose integrity is governed by complex processes such as DNA methylation and an array of histone modifications.^1^, ^2^ These chromatin modifications exert a profound influence on a myriad of cellular and developmental events, functioning as quintessential regulators of both gene and genomic landscapes.

Chromatin architecture is orchestrated through the strategic assembly of nucleosomes, which are composed of histone octamers—formed by histone proteins H2A, H2B, H3, and H4—around which superhelical DNA is wrapped.^1^, ^2^ An extensive spectrum of covalent modifications, including but not limited to acetylation, methylation, phosphorylation, and *O*‐GlcNAcylation, have been documented on both histone amino‐terminal tails and globular histone cores.^3^, ^4^ Furthermore, variations in histone methylation—mono‐, di‐, and tri‐methylation—have been discerned to have distinct functional roles.^3^, ^4^ DNA is also susceptible to diverse modifications; in mammalian cells, the most prevalent include 5‐methylcytosine (5mC) and its oxidative derivatives—5 hydroxymethylcytosine (5hmC), 5 formylcytosine, and 5 carboxylcytosine—all of which contribute to the intricate regulatory network governing gene and genome function.^5^, ^6^ It is evident that these chromatin modifications are pivotal in modulating a plethora of biological activities, encompassing gene transcription and expression, DNA replication, DNA damage repair, and DNA recombination. Emerging evidence implicates that malignant transformation is associated with aberrant epigenetic landscapes and altered posttranslational modifications of histones. Importantly, these epigenetic changes are often fueled by metabolic intermediates, thereby establishing a feedback loop with cancer metabolism.

Metabolic reprogramming constitutes a hallmark of cancer pathobiology, significantly influencing a diverse range of abnormal metabolic pathways, including glycolysis, lipogenesis, and amino acid biosynthesis and catabolism, all of which have ramifications for cancer progression and treatment responsiveness.^7^, ^8^, ^9^, ^10^, ^11^, ^12^, ^13^ Various metabolic enzymes catalyze these processes, generating a plethora of intracellular metabolites from extracellular nutrients such as glucose, lipids, amino acids, and vitamins. Notably, the regulatory circuitry of cellular metabolism can be tuned through the modulation of metabolic genes, which respond to both extrinsic and intrinsic nutrient cues. Additionally, the activities of chromatin can be altered via changes in metabolic enzymes or metabolites. Recent studies have illuminated that some metabolic enzymes undergo nuclear translocation in neoplastic tissues and serve dual functional roles: metabolic catalysis and the regulation of gene transcription pertinent to tumor cell proliferation.^13^, ^14^, ^15^, ^16^

Elucidating the nuclear functions of metabolic signaling—including both metabolites and metabolic enzymes, collectively termed as “nuclear metabolic signaling”—promises not only to unveil novel therapeutic targets but also to inspire innovative treatment strategies targeting the dual‐function metabolic enzymes or the intricate metabolism‐gene transcription axis. These potential targets are implicated not merely in cellular metabolism but also in an array of nuclear processes that influence tumoral proliferation, such as gene transcription, DNA damage repair, and chromatin modification. Hence, the identification and characterization of these nuclear roles are of paramount importance for advancing cancer research and therapeutic modalities.

In this review, we aim to provide a comprehensive analysis of metabolic signaling within the nuclear compartment. We will examine the multifaceted functions of these signaling components in the nuclear environment, their impact on other nuclear biological processes, and the ensuing therapeutic strategies and clinical applications.

## 2 NUCLEAR METABOLIC DYSREGULATION IN CANCER

Metabolic reprogramming is increasingly acknowledged as a salient hallmark of cancer pathobiology. Such reprogramming culminates in an overabundance of metabolites and metabolic enzymes, which serve not only as nutritional substrates but also infiltrate the nuclear compartment to modulate gene expression, thereby fueling cancer cell proliferation. Consequently, elucidating the intricacies of nuclear metabolic signaling and its regulatory mechanisms has become an imperative avenue of inquiry. We will introduce roles of nuclear metabolism in cancer growth and mechanisms of nuclear metabolic dysregulation in following discussion. It includes various modifications which affect the epigenetic characteristics and nuclear metabolic enzymes which usually present in the cytoplasm.

### 2.1 Role of nuclear metabolism in cancer cell growth and proliferation

The pathogenesis of cancer is an intricate tapestry woven from multiple contributing factors, among which metabolic anomalies stand as pivotal elements. Within this context, nuclear metabolic signaling represents an indispensable component of cellular metabolism, orchestrating a myriad of cellular functions essential for growth and proliferation. Metabolites that encroach upon the nuclear domain in tumor cells serve multifaceted roles—acting as cofactors, molecular donors for modifications, or agents that either potentiate or attenuate biochemical activities, thereby exerting a profound influence on the epigenetic framework.

For instance, the S‐adenosylmethionine (SAM) to S‐adenosylhomocysteine (SAH) ratio serves as a crucial regulatory axis, modulating the activities of an array of methyltransferases, and thereby overseeing methylation patterns across DNA, RNA, and histones.^17^, ^18^, ^19^ Similarly, acetyl coenzyme A and nicotinamide adenine dinucleotide (NAD^+^) play regulatory roles in histone acetylation, thereby adding nuanced layers to an already complex epigenetic landscape.^20^, ^21^ Jumonji C domain‐containing histone demethylases (JHDMs) are activated by α‐ketoglutarate (α‐KG) and inhibited by 2‐hydroxyglutarate (2HG), succinate, and fumarate esters. α‐KG serves as a cosubstrate, facilitating the removal of methyl groups from histones, thereby generating succinate and formaldehyde as byproducts.^22^ Furthermore, dysregulation in mRNA methylation profiles has been shown to wield influence over the fate of tumor cells by selectively modulating genes implicated in diverse malignancies.^23^ Interestingly, recent report suggests that the translation process can also occur within the nucleus and regulate tumor growth.^24^ Amino acids, mature transfer RNA (tRNA), messenger RNA (mRNA), and ribosome which are required for the translation process, are found to appear in the nucleus. Zou et al.^25^ demonstrate numerous oncoproteins that are preferentially translated in the nucleus (e.g., TGFβ2 and NMP1), and high mRNA level in the nucleus increase protein pression and promote tumorigenesis.

Significantly, these events are metabolically driven, requiring substrates or cofactors such as SAM, acetyl coenzyme A, α‐KG, NAD^+^, ATP, and succinate. Alterations in the patterns of histone or DNA/RNA modification are frequently correlated with oncogenesis. Hence, nuclear metabolic signaling plays an instrumental role in the promotion of cancer cell growth and proliferation by modulating an array of oncogenes. Comprehensive insights into the role of nuclear metabolism could pave the way for the development of innovative and more efficacious therapeutic paradigms, thereby enhancing the landscape of cancer treatment.

### 2.2 Mechanisms underlying nuclear metabolic dysregulation in cancer

Nuclear metabolic signaling orchestrates gene expression by influencing a spectrum of molecular mechanisms, encompassing epigenetic modifications, RNA/protein posttranslational modifications, and the moonlighting functions of metabolic enzymes within the nuclear milieu. These represent the predominant mechanisms through which nuclear metabolic signaling exerts its regulatory influence on gene expression.

#### 2.2.1 Metabolic control of epigenetic modification

##### Histone methylation

Histone methylation, a prevalent posttranslational modification regulated by histone methylases (HMTs), is ubiquitously identified across mammalian species and demonstrates a significant association with oncogenesis. This modification principally involves the methylation of lysine (K) or arginine (R) residues, predominantly localized at the amino termini of core histones H3 and H4, including but not limited to the loci H3K4, H3K9, H3K36, H3K79, and H4K20.^26^ A spectrum of methylation states—mono‐, di‐, and trimethylation (notated as me1, me2, and me3, respectively)—can be engendered at the lysine residues by the enzymatic activity of histone lysine methyltransferases.^27^ Conversely, the methylation of arginine residues is orchestrated by arginine methyltransferases, culminating in either mono‐ or dimethylated arginine derivatives.^28^

**FIGURE 1:**
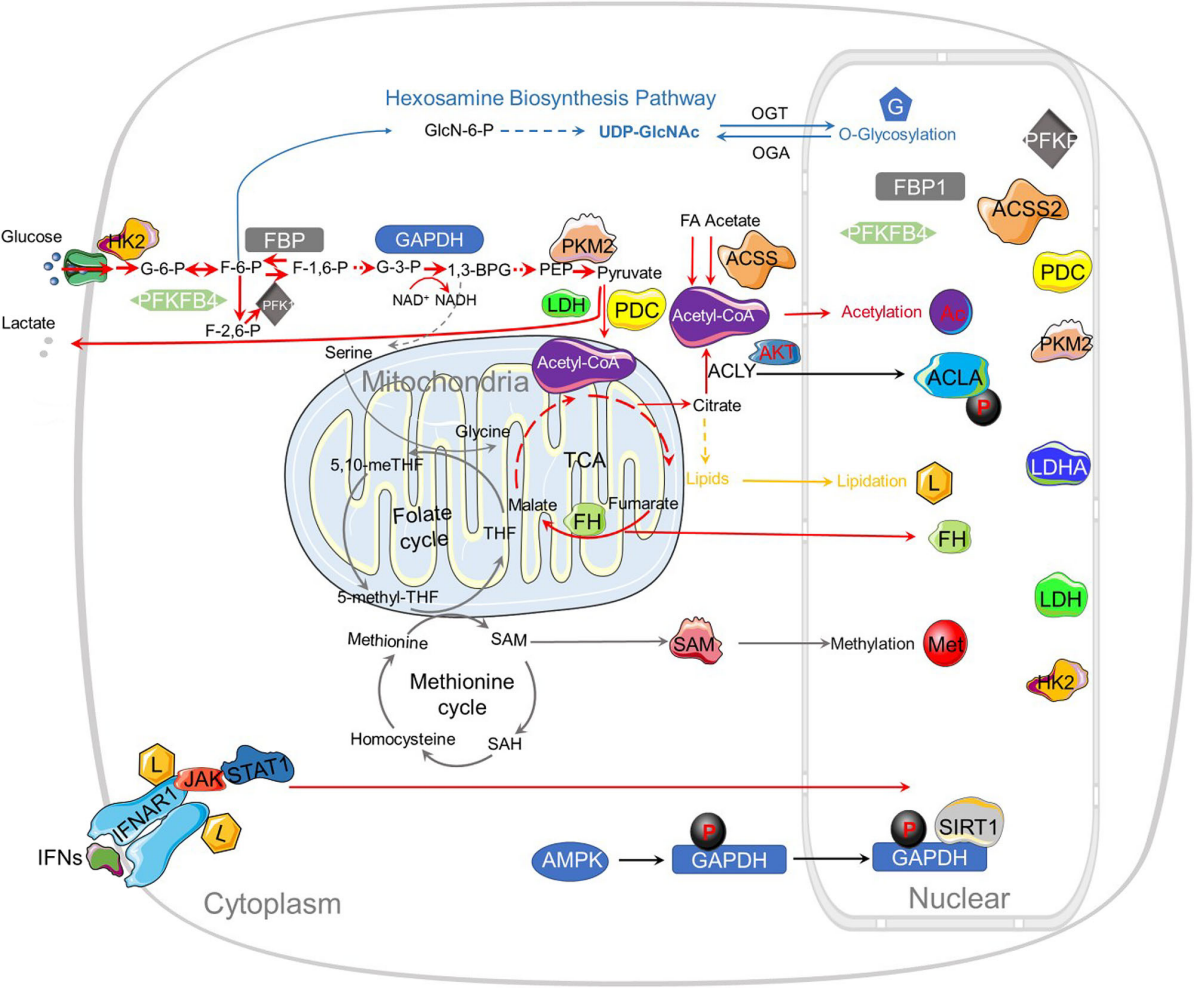
Metabolic reprogramming affects the abundance of key metabolites and metabolic enzymes in the nucleus. Within the tumorigenic microenvironment, aberrant metabolic activity amplifies metabolic flux, thereby accelerating the turnover rates of molecular entities and augmenting the concentration of intranuclear metabolites. Elevated levels of metabolic enzymes stand as a primary determinant in shaping the metabolic landscape of cancer cells. These enzymes translocate into the nuclear compartment, where they contribute to the augmented abundance of pivotal metabolites and enzymes. These, in turn, modulate gene expression through a diverse array of molecular modifications including methylation (denoted as Met), acetylation (Ac), O‐glycosylation (G), and lipidation (L). Nuclear metabolic enzymes are emphasized within the figure for clarity.

**FIGURE 2:**
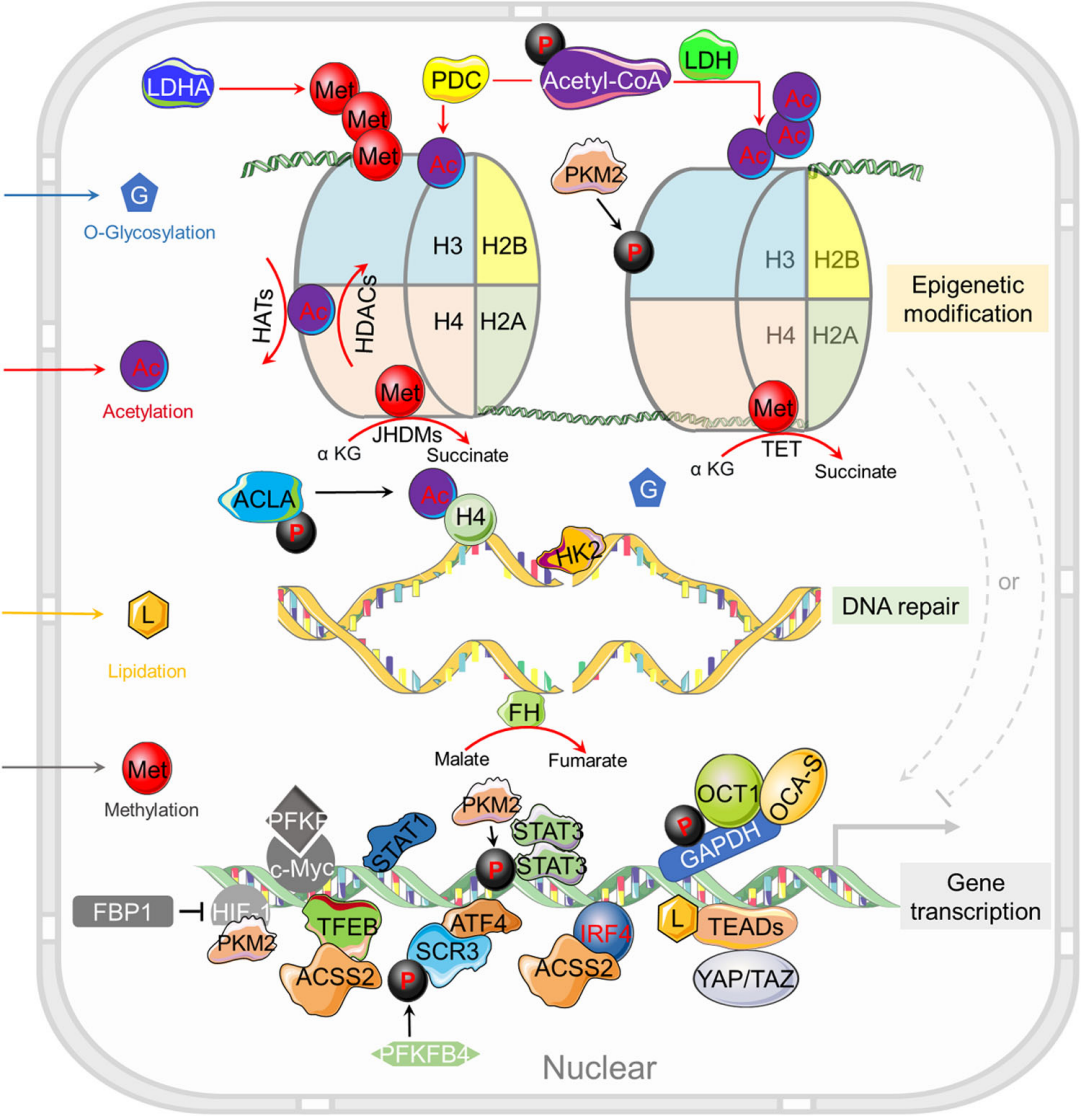
How metabolic alterations impact nuclear gene regulation in cancer. Abnormal metabolic activities in tumor cells facilitate alterations in chromatin or protein modifications such as methylation (Met), acetylation (Ac), O‐glycosylation (G), and lipidation (L), mediated through the control of key metabolites like SAM, acetyl‐CoA, UDP‐GlcNAc, and lipids. Nuclear metabolic enzymes, originating from specific metabolic pathways, exert their influence on chromatin architecture, DNA repair mechanisms, and gene transcription through diverse regulatory avenues. Perturbations in epigenomic stability or genomic fidelity can reciprocally affect the transcriptional profile of metabolic genes, thus establishing a dynamic metabolism‐epigenetics nexus that enables tumor cells to adapt and thrive in variable environments.

SAM serves as the universal methyl donor, predominantly synthesized from methionine via the one‐carbon metabolism pathway, facilitated by methionine adenosyl transferases.^29^ Methylation events are constrained in a cellular milieu characterized by low SAM concentrations.^5^ The subsequent demethylation of SAM yields SAH, a potent inhibitor of all methyltransferase activity.^17^, ^30^ Consequently, the intracellular SAM:SAH ratio serves as a significant modulator of global methylation profiles. Importantly, cellular SAM and SAH concentrations are conspicuously attenuated under conditions of methionine scarcity.^17^ Depletion of methionine in the methionine cycle reverberates through alterations in the SAM/SAH ratio, thereby influencing histone methylation profiles^17^ (Figures 1 and 2).

It is of considerable note that deletions involving methylthioadenosine phosphorylase (MTAP)—a pivotal enzyme in the methionine salvage pathway—have been implicated in multiple cancer phenotypes.^30^ A compromised MTAP expression precipitates a reduction in intracellular methionine levels, engendering a heightened dependency of malignant cells on exogenous methionine.^30^ Remarkably, in juxtaposition with normal cells, neoplastic cells manifest an elevated consumption of methionine, a metabolite of critical importance in the proliferation of certain cancer subtypes.^30^, ^31^ As early as 1958, a correlation between dietary methionine levels and tumorigenesis was reported. Specifically, the rate of tumor growth markedly decelerates in animal models subjected to methionine‐deficient diets.^32^ Contemporary research further corroborates that dietary methionine restriction can not only attenuate tumor proliferation but also potentiate the efficacy of chemotherapeutics such as 5‐FU in patient‐derived xenograft models of colorectal cancer.^33^ Additionally, methionine restriction has been shown to downregulate H3K4 methylation levels, thereby modulating the transcriptional activity of cancer‐associated genes like Myc, MAPK, and AKT, as evidenced through chromatin immunoprecipitation followed by sequencing (ChIP‐seq) analyses.^17^ H3K4 methylation, a cardinal chromatin modification, is intricately associated with gene activation. The trimethylated form, H3K4me3, preferentially recruits transcriptional activators to gene promoters whilst precluding the association of transcriptional repressors, such as nucleosome remodeling and deacetylase complexes.^34^, ^35^, ^36^ Within the specific context of pancreatic neoplasia, elevated levels of H3K4 trimethylation (H3K4me3) have been localized at the cd274 promoter region, thereby amplifying the transcription of PD‐L1 and facilitating immune evasion by the tumor.^37^

Defects in serine metabolism exert a consequential impact on the levels of SAM in neoplastic cells. Serine serves as a principal donor of one‐carbon units to the folate cycle, an integral component of one‐carbon metabolism^38^ (Figure 1). Within the folate cycle, serine undergoes a transformative process to yield glycine and one‐carbon moieties that subsequently conjugate with tetrahydrofolate (THF), culminating in the synthesis of 5,10‐methylene tetrahydrofolate (MTHF). This compound is subsequently metabolized into 5‐methyltetrahydrofolate (5‐MTHF) via the enzymatic activity of 5‐methylene THF reductase (MTHFR), thereby regenerating THF.^39^, ^40^ The methyl group liberated from 5‐MTHF engages in a binding interaction with homocysteine (HCys), resulting in the synthesis of methionine and instigating the methionine cycle.^29^, ^41^ Consequently, serine bioavailability critically influences cellular SAM concentrations and overarching methylation dynamics. Furthermore, intracellular serine concentrations can be augmented either via the biosynthetic conversion of glycolytic byproduct 3‐phosphoglycerate or through direct uptake. Notably, phosphoglycerate dehydrogenase, the cardinal enzyme in the serine biosynthetic pathway, is observed to be amplified in specific malignancies, such as breast cancer and melanoma.^42^

Histone demethylases (HDMs), colloquially termed “erasers,” possess the capability to expunge methylation moieties from histones. Importantly, these enzymes leverage a variety of metabolites as cofactors in the aforementioned biological cascade (Figure 1). Two primary categories of HDMs have been identified: lysine‐specific protein demethylases 1 (LSD1) and JHDMs. JHDMs are allosterically activated by α‐KG and inhibited by 2HG, succinic acid, and fumarate. α‐KG, serving as a cosubstrate, facilitates the removal of methyl groups from histones through JHDM‐mediated reactions, subsequently yielding succinate and formaldehyde.^22^ Typically, α‐KG is biosynthetically derived from isocitrate via isocitrate dehydrogenase (IDH) within the context of the tricarboxylic acid (TCA) cycle. Intriguingly, mutations in IDH have been implicated in a spectrum of malignancies, including acute myeloid leukemia (AML), glioblastoma, and T‐cell lymphoma.^39^ Mutations in IDH isoforms (IDH1 and IDH2) abrogate the enzymatic synthesis of α‐KG, while concurrently engendering the accumulation of the oncometabolite 2HG. Subsequently, α‐KG‐dependent enzymes, such as HDMs, are competitively inhibited by the structurally analogous metabolite (R)−2‐hydroxyglutarate [(R)−2HG]. 2‐Oxoglutarate serves as an integral intermediate of the TCA cycle and constitutes a pivotal cofactor for a diverse array of enzymes.^40^, ^42^, ^43^

##### DNA methylation

The mammalian genome is characterized by a high prevalence of CpG DNA methylation, with approximately 60−80% of CpG dinucleotides being methylated, excluding CpG islands—CpG‐rich domains that are predominantly unmethylated and encapsulate the promoters for up to 60% of genes.^44^, ^45^, ^46^ It is noteworthy that genomic hypomethylation of CpG loci is typically associated with elevated gene expression, while hypermethylation, particularly within promoter or enhancer regions, often culminates in transcriptional silencing.^47^, ^48^ Distinct from normal cellular states, cancerous cells exhibit aberrant methylation profiles, characterized by hypomethylation in regions with low CpG density and selective hypermethylation of CpG islands.^42^ This methylation landscape is perpetuated across generations through the enzymatic action of DNA methyltransferases (DNMTs) during DNA replication. All three isoforms of DNMTs—DNMT1, DNMT3A, and DNMT3B—are found to be overexpressed in various malignancies.^49^ SAM, a one‐carbon donor derived from the methionine cycle, serves a dual role in both DNA and histone methylation. Consequently, perturbations in methionine metabolism can profoundly influence DNA methylation via modulating SAM availability. In colorectal cancer cells, for example, de novo ATP production, facilitated by serine metabolism, augments the conversion of methionine to SAM within the methionine cycle. Serine deficiency subsequently elevates the methionine‐to‐SAM ratio, attenuating the transfer of methyl groups to DNA.^50^ Recent investigations have demonstrated that inhibited protein kinase C activity in both de novo and therapy‐induced neuroendocrine prostate cancer (NEPC) activates mTOR, thereby upregulating serine biosynthesis and increasing SAM levels to facilitate NEPC development.^38^ Additionally, dysregulated tumor methionine metabolism has been implicated in T‐cell modulation. Integrative omics studies have posited that in hepatocellular carcinoma, T‐cell exhaustion is associated with alterations in methionine recycling, evidenced by elevated levels of 5‐methylthioadenosine and SAM, which contribute to T‐cell dysfunction in vitro.^49^

DNA methylation represents a dynamic equilibrium between two antithetical processes: DNA methylation and DNA demethylation. The ten‐eleven translocation (TET) family of proteins and thymine DNA glycosylase function as catalytic facilitators of DNA demethylation.^51^, ^52^ Fe^2+^, O2, and α‐KG serve as pivotal cofactors in TET‐mediated reactions^53^, ^54^ (Figures 1 and 2). Neoplastic cells harboring mutations in metabolic enzymes, such as IDH, succinate dehydrogenase, and fumarate hydratase (FH), are prone to the intracellular accumulation of TCA cycle intermediates, including 2HG, succinate, and fumarate. These metabolites act as inhibitory agents against TET activity, thereby exacerbating tumorigenesis through increased DNA methylation.^55^ Moreover, gene silencing through promoter hypermethylation plays a modulatory role in cancer metabolism. For instance, fructose‐1,6‐bisphosphatase (FBP1), the rate‐limiting enzyme in gluconeogenesis, catalyzes the conversion of fructose‐1,6‐diphosphate to fructose‐6‐phosphate (F6P).^56^ The hypermethylation of the FBP1 promoter results in downregulation of its expression, a phenomenon observed in both lung and breast cancers.^56^, ^57^

##### Histone acetylation

Histone acetylation constitutes a ubiquitous, reversible posttranslational modification mediated by two distinct classes of enzymes: histone acetyltransferases (HATs) and histone deacetylases (HDACs) (Figure 2). HATs orchestrate the covalent attachment of an acetyl moiety to a lysine residue, whereas HDACs facilitate the removal of the acetyl group. The dysregulation of these enzymes is intimately implicated in the pathogenesis of tumorigenesis.^58^ Acetylation alters the electrostatic attributes and the local microenvironment of chromatin, thereby modulating its structure to a more accessible state, facilitating the recruitment of transcription factors, and expediting gene transcription. Conversely, deacetylation operates to restrain transcriptional activity.^59^ Intriguingly, neoplastic cells typically manifest elevated levels of histone acetylation, aligning with their heightened transcriptional activities.

The rate of enzymatic acetylation and deacetylation is contingent upon the availability of acyl group donors. Acetyl‐coenzyme A (acetyl‐CoA) serves as a principal substrate for the acetylation catalyzed by HATs (Figure 1). The metabolic substrates—pyruvate, citrate, and acetate—are converted into acetyl‐CoA through the enzymatic activities of the pyruvate dehydrogenase complex (PDC), ATP citrate lyase (ACLY), and acetyl‐CoA synthetase short‐chain family member (ACSS), respectively.^60^ Additionally, acetyl‐CoA can be synthesized through the β‐oxidation of fatty acids and other nutrient metabolism pathways, such as those involving amino acids and ketones. Hence, the global histone acetylation state is potentially influenced by the cellular abundance of acetyl‐CoA.

In a Ca^2+^‐responsive mechanism involving the NFAT1 transcription factor, acetyl‐CoA has been observed to promote glioblastoma cell migration and adhesion to the extracellular matrix.^61^ Elevated intracellular concentrations of acetyl‐CoA lead to an increase in intracellular Ca^2+^ levels, thereby facilitating the nuclear translocation of Ca^2+^‐dependent NFAT1. The transfer of acetyl‐CoA from the mitochondrial matrix to the cytosol is mediated by citrate, which is subsequently cleaved back into oxaloacetate and acetyl‐CoA by ACLY.^62^ Given the pivotal role of acetyl‐CoA in histone acetylation, the downregulation or loss of ACLY/ATPCL function precipitates a reduction in histone acetylation levels.^62^ Therefore, cellular metabolism is intricately entwined with epigenetic modifications, specifically histone acetylation.

Notably, histone acetylation serves as a critical facilitator of proficient DNA repair mechanisms. It aids in the recruitment of specialized repair proteins and ensures unimpeded access to double‐strand break (DSB) loci for the DNA repair machinery.^63^ Thus, the modulation of histone acetylation has far‐reaching implications for cellular integrity and function.

Recent investigations have elucidated those posttranslational modifications of specific nuclear genes, including but not limited to ACSS, ACLY or ACLA, and PDC, exhibit a nuanced interplay with HATs or nuclear transcription factors, thus potentially influencing chromatin regulation^64^ (Figure 2). Oncogenic signaling pathways have been demonstrated to induce ACLY‐dependent synthesis and utilization of acetyl‐CoA in malignancies, and empirical evidence substantiates the existence of a positive correlation between global histone acetylation levels and phosphorylated AKT at Ser473 (pAKT–S473) in gliomas and prostate cancers.^20^

ACLY serves as a unique substrate that is sensitive to mechanistic target of rapamycin complex 2 (mTORC2), which subsequently modulates ACLY activity to enhance ACSS2‐mediated synthesis of acetyl‐CoA from acetate, thereby augmenting histone acetylation.^65^ Notably, AKT‐mediated phosphorylation of ACLY at the S455 site enhances its enzymatic activation.^66^ This activated AKT is instrumental in escalating acetyl‐CoA production and promoting acetylation of histones H3 and H4 via the phosphorylation of ACLY.^20^ Moreover, phosphorylated nuclear ACLY is responsive to DNA damage signals and fortifies homologous recombination DNA repair mechanisms through the orchestrated spatiotemporal synthesis of nuclear acetyl‐CoA, thereby modulating H4 acetylation at DSB sites.^64^

Furthermore, ACSS2 plays a discernible role in furnishing acetyl‐CoA requisite for nuclear histone acetylation.^67^ Under low‐glucose conditions, ACSS2, when phosphorylated by AMP‐activated protein kinase (AMPK), engages in interactions with transcription factor EB (TFEB) and repositions itself at gene promoters to initiate lysosomal biogenesis and autophagy as an adaptive response.^67^ In myeloma cells sourced from obese patients, ACSS2 is regulated by adipocyte‐secreted angiotensin II, where it stabilizes interferon regulatory factor 4 (IRF4) through acetylation, thereby upregulating IRF4‐controlled gene expression.^67^

Moreover, in the realm of prostate cancer, nuclear PDC has been shown to induce acetylation at histone 3 lysine 9 (H3K9) in the promoter regions of lipid‐associated genes, such as ACLY and squalene epoxidase, thereby facilitating lipogenesis and cellular proliferation.^68^ In a parallel mechanism, mitochondrial PDC provides cytosolic citrate as a substrate for lipid synthesis to sustain cellular metabolism.^68^ In HeLa cells, an association between nuclear PDC and the aryl hydrocarbon receptor has been reported.^69^ Additionally, it has been corroborated that the growth of breast cancer cells exhibiting high ACSS2 expression can be mitigated by a small‐molecule inhibitor targeting ACSS2.^70^

HDACs are zinc ion‐dependent enzymes and display subtype‐specific roles across a plethora of tumors. Elevated levels of HDAC1 have been reported in gastric and prostate cancers, while HDAC2 expression is abundant in colon, cervical, and gastric cancers.^71^ Interestingly, fatty acid hydrolysis can directly inhibit HDAC1 and HDAC2a by generating butyrate and β‐hydroxybutyrate (β‐HB).^72^ Another cadre of HDACs is constituted by the sirtuin family, notably SIRT1 and SIRT2. Elevated levels of NAD^+^ modulate and amplify the enzymatic activity of the sirtuin family, counteracting the inhibitory effect of nicotinamide (NAM), a precursor to NAD^+^.^73^

##### Histone *O*‐glycosylation

Nucleotide sugars, which function as substrates for glycosylation processes, are generated through the hexosamine biosynthetic pathway (HBP). A salient substrate implicated in both *N*‐ and *O*‐linked glycosylation is uridine diphosphate N‐acetylglucosamine (UDP‐GlcNAc).^74^, ^75^ The *O*‐GlcNAcylation process specifically entails the singular O‐linked attachment of GlcNAc moieties to serine and threonine residues on proteins. This dynamic posttranslational modification is orchestrated by two pivotal enzymes: O‐GlcNAc transferase (OGT) and O‐GlcNAcase, which catalyze the addition and removal of O‐GlcNAc modifications, respectively.^76^

Dysregulated O‐GlcNAcylation has been implicated in the modulation of signal transduction pathways, gene expression profiles, and metabolic processes, cumulatively contributing to oncogenic transformation and cancer progression.^77^, ^78^, ^79^ Notably, an augmented level of O‐GlcNAcylation is frequently observed in various malignancies, and targeted suppression of OGT has been demonstrated to mitigate cancer growth.^80^, ^81^, ^82^ Histone O‐GlcNAcylation is a ubiquitous phenomenon that engages in intricate crosstalk with other posttranslational modifications, such as methylation, acetylation, phosphorylation, and ubiquitination, to govern gene expression^83^ (Figures 1 and 2).

In specific cancer types such as breast and colon cancer, the knockdown of OGT engenders a significant diminution in H3K27me3 levels and in the protein stability of Enhancer of Zeste Homolog 2 (EZH2), without influencing the concentrations of H3K27 demethylases, UTX, and JMJD3.^84^ Elevated O‐GlcNAcylation in tumorigenic cells has also been shown to attenuate the intracellular levels of α‐KG. This in turn affects the α‐KG‐dependent prolyl hydroxylation of hypoxia inducible factor‐1 (HIF‐1), thereby stabilizing HIF‐1 and modulating target gene expression, including that of GLUT1. This cascade of events serves to accelerate glycolysis, suggesting that tumors employ a feed‐forward metabolic signaling loop mediated via the HBP to sustain their metabolic idiosyncrasies.^80^

OGT itself is subject to direct phosphorylation at the Thr 444 site by AMPK. This phosphorylation disrupts OGT's chromatin interaction and attenuates histone H2B O‐GlcNAcylation, albeit without exerting a direct influence on OGT's enzymatic capabilities.^85^, ^86^ O‐ or N‐GlcNAcylation also contribute to other oncogenic mechanisms, such as the upregulation of lipid biosynthesis via modulation of SCAP/SREBP.^12^, ^87^

Beyond histones, a repertoire of epigenetic regulators are also susceptible to O‐GlcNAcylation. For instance, OGT forms a complex with the methylcytosine dioxygenases TET1, TET2, and TET3—each of which is highly O‐GlcNAcylated. In a reciprocal fashion, these TET proteins anchor OGT to chromatin, where they regulate the conversion of 5mC to 5hmC, a process intricately modulated by both OGT and O‐GlcNAcylated TET proteins.^79^, ^83^

##### Protein lipid modification

Lipid moieties, characterized by their hydrophobic groups, can be covalently affixed to proteins via an array of linkages, including amide, ester, thioester, or thioether bonds. Such lipid‐modified proteins fulfill multifaceted roles, encompassing the regulation of protein functionality, subcellular localization, and intricate interactions with other cellular entities, such as proteins, lipids, cofactors, and nucleic acids.^88^, ^89^, ^90^, ^91^, ^92^, ^93^ Protein lipid modifications are generally categorized into three predominant types: S‐ or N‐palmitoylation, N‐myristoylation, and S‐prenylation. Specifically, S‐palmitoylation and N‐myristoylation typically target cysteine thiols (S‐acylation) and the amine groups of glycine residues (N‐myristoylation), respectively. In contrast, S‐prenylation involves the formation of thioether bonds between thiols on cysteine residues and isoprenoid groups. These lipid modifications enhance the hydrophobic character of proteins, thereby facilitating their affinity for membrane interaction or nuclear translocation. For example, the estrogen receptor (ER) α undergoes a translocation from the plasma membrane to the nucleus subsequent to its de‐S‐palmitoylation.^94^ Additionally, palmitoylation has been demonstrated to stabilize transcriptional enhanced associate domains and augment their interactions with the transcriptional coactivators Yes‐associated protein and Tafazzin^95^ (Figure 2).

The cell surface receptor interferon alpha receptor 1 (IFNAR1) serves as a ligand for type I interferons (IFNs), thereby facilitating the activation of Janus kinase (JAK) and signal transducer and activator of transcription (STAT) to modulate the transcription of downstream genes via IFN‐stimulated response elements (Figures 1 and 2). However, the absence of palmitoylation in IFNAR1 leads to a compromised STAT2 activation, which consequentially hampers STAT1 activation and nuclear translocation, culminating in the inhibition of IFN‐α‐activated gene transcription.^96^ Recent scholarly contributions have revealed that ZDHHC19 (Z19) functions as a palmitoyl acyltransferase that modulates STAT3 in lung cancer. Elevated expression levels of Z19 have been correlated with increased nuclear STAT3 expression in cancer patients. Furthermore, the SRC Homology 2 (SH2) domain of STAT3 undergoes posttranslational S‐palmitoylation, thereby stimulating STAT3 transcriptional activation and dimerization. Fatty acids have also been identified to potentiate STAT3 activation via palmitoylation, in synergy with cytokine activation.^97^

Oct4 (POU5F1), a transcription factor, exists in variants including Oct4A, Oct4B, and Oct4B1.^98^ Palmitoylation orchestrated by ZDHHC17 (Z17) is critically implicated in shielding Oct4A from lysosomal degradation, thereby preserving its protein stability.^99^ This modification also facilitates the assembly of transcription factors, such as SOX4 and Oct4A, in the SOX2 enhancer region, thereby sustaining the stemness attributes of glioma stem cells.^99^ Analogous to palmitoylation, myristoylation serves as a regulatory mechanism for membrane anchoring, protein–protein or protein–lipid interactions, while concurrently inhibiting protein degradation and gene transcription.^90^, ^100^, ^101^

EZH2, a lysine methyltransferase, orchestrates DNA methylation to repress gene transcription.^102^, ^103^ Within the context of oncogenic signaling, STAT3 is identified as a bona fide substrate for EZH2.^104^ The N‐terminal glycine of EZH2 undergoes myristoylation in lung cancer cells, thereby instigating liquid–liquid phase separation (LLPS). This process enables the compartmentalization of STAT3 by EZH2's LLPS and concurrently activates STAT3 signaling.^105^ Myristoylation of EZH2 also augments its interaction with STAT3, thereby enhancing STAT3 Y705 phosphorylation and transcriptional activity, which ultimately propels lung cancer progression.^105^

Wilms' tumor 1 protein undergoes a functional transformation from a transcriptional activator to a repressor via its interaction with the transcriptional corepressor BASP1.^106^ Within the nuclear milieu, myristoylated BASP1 associates with phosphatidylinositol 4,5‐bisphosphate (PIP2), facilitating the recruitment of PIP2 to the promoter regions of target genes.^98^This intricate interaction between BASP1 and PIP2 catalyzes the recruitment of HDAC1 to gene promoters, thereby effectuating transcriptional repression.^98^

##### RNA modification

**FIGURE 3:**
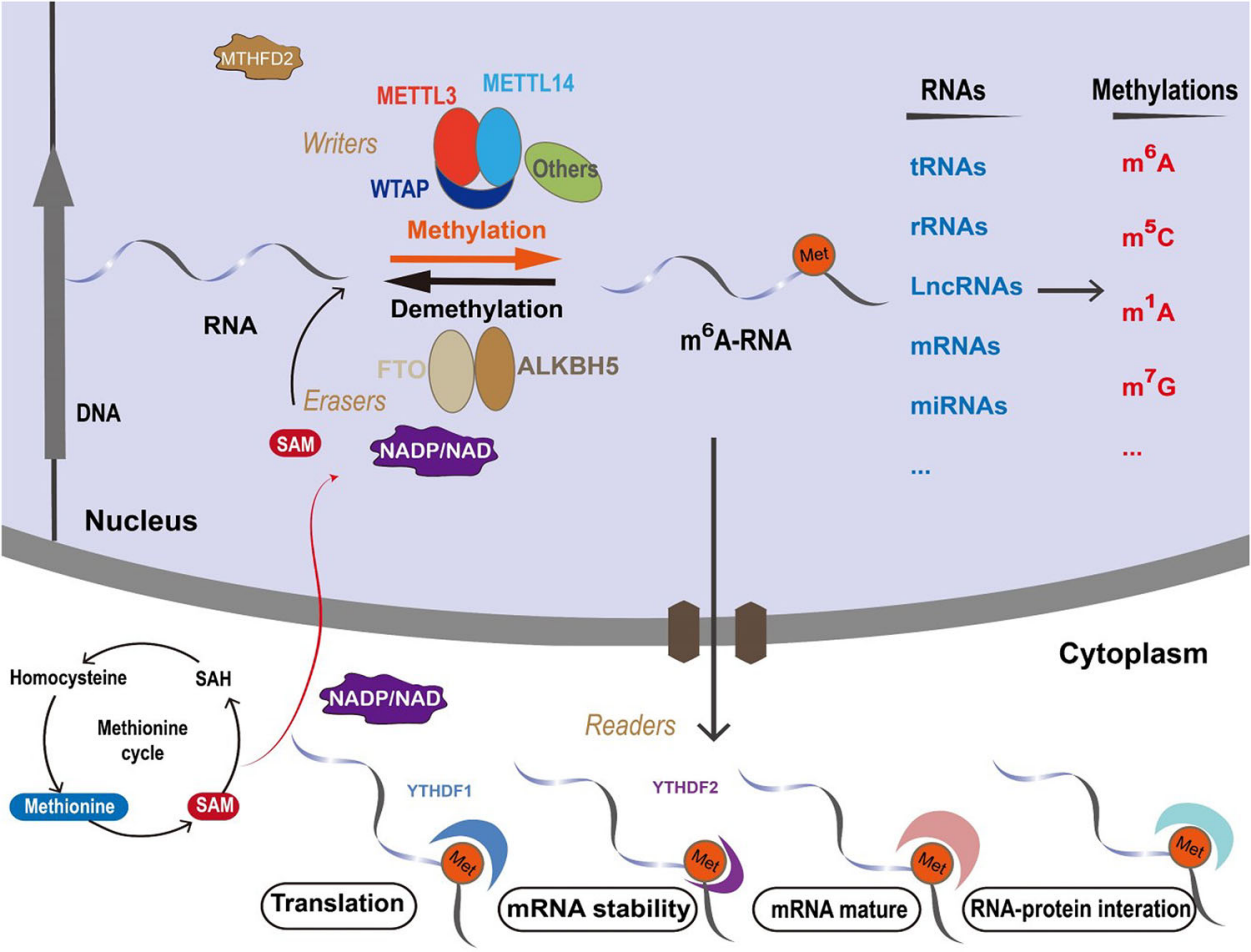
The interplay between nuclear metabolic signaling and RNA methylation. Nuclear metabolic signaling components, including SAM, methionine, NADP, and NAD, impact an array of RNA methylation modifications, such as m6A, m5C, m1A, and m7G, among others. These modifications can be enzymatically installed or removed by “writers” (methyltransferases) and “erasers” (demethylases), and are discerned by their respective “readers” (e.g., YTHDF1 and 2 for m6A modification). In the cytoplasmic milieu, m6A modifications and their regulatory elements partake in mRNA translation, stability, maturation, and RNA–protein interactions, collectively contributing to the advancement of oncogenesis.

The burgeoning advances in RNA research have culminated in the identification of an expansive repertoire of RNA modifications, enabled by the advent of highly specialized, quantitatively accurate, and sensitively precise investigative methodologies. To this point, an array of over 170 discrete modifications have been meticulously characterized within various RNA classes, including tRNAs, ribosomal RNAs (rRNAs), long noncoding RNAs (lncRNAs), mRNAs, microRNAs (miRNAs), among others.^23^ Of particular prominence is the substantial body of research focusing on mRNA methylation, comprising modifications such as m6A, m5C, m1A, and m7G. RNA methylation serves as a burgeoning category of epigenetic modification with significant roles in modulating a myriad of biological processes, including but not limited to gene expression, RNA stability, translational control, and intricate RNA–protein interactions.^107^, ^108^, ^109^, ^110^, ^111^ In the medical landscape, the study of m6A modifications has garnered considerable attention for its pertinence to a multitude of diseases, most notably various oncological conditions (Figure 3). This amplified focus emanates from observations that both m6A and its associated regulatory machinery exhibit dysregulation across different cancer types.^112^

As a pivotal regulatory modality, RNA methylation exerts influence over an assortment of metabolic pathways, thereby shaping the trajectory of tumor initiation and subsequent progression. Recent scholarly investigations suggest that nuclear metabolic signaling has the potential to fine‐tune RNA methylation levels through a variety of mechanistic avenues, offering profound implications for the dynamics of cancer development. Analogous to DNA or histone methylation, the methyl groups pertinent to RNA methylation are derived from SAM, implicating that both the methionine and folate cycles exert an intricate regulatory influence on RNA methylation levels.^18^, ^19^, ^113^ Empirical evidence demonstrates that nutritional regimens characterized by restriction, high‐fat, or high‐sugar content can modulate m6A levels through their impact on FTO (a demethylase or “eraser”) and METTL3 (a methyltransferase or “writer”).^113^ Furthermore, dietary restriction has been shown to precipitate an increase in hypothalamic FTO levels in rat models.^113^ Concurrently, methionine and SAM have been documented to upregulate the expression of METTL3, thereby instigating augmented c‐Myc expression and Avpr2 mRNA m6A modification. Such alterations further activate the c‐Myc and cAMP signaling pathways.^19^ An additional seminal study revealed that curtailing dietary methionine intake in murine models led to a marked reduction in levels of l‐cysteine hydropersulfide (LCYH), SAM, SAH, GSH, and l‐methionine in tumor tissues. This diminution subsequently resulted in a significant decrement in m6A methylation and expression of immune checkpoints, including PD‐L1 and V‐domain Ig suppressor of T cell activation (VISTA).^18^ These collective findings advance the notion that targeting methionine metabolism as a modulatory tactic for m6A levels might present a novel therapeutic avenue in cancer treatment. Methylenetetrahydrofolate dehydrogenase 2 (MTHFD2) is a mitochondrial enzyme intricately involved in the folate cycle; it modulates m6A methylation and impacts the clinical course of renal cell carcinoma. MTHFD2 enhances METTL3‐dependent methylation of HIF‐2α mRNA, subsequently promoting HIF‐2α expression. In a reciprocal manner, HIF‐2α binds to the promoter of the MTHFD2 gene, culminating in elevated MTHFD2 levels, thereby perpetuating a positive feedback loop contributing to metabolic reprogramming and tumoral progression.^114^

Apart from the methionine and folate cycles, additional metabolic pathways and metabolites also hold the capacity to modulate m6A modifications. Under hypoxic conditions, HIF‐1α governs the transcription of YTHDF1, a recognized m6A reader, by directly engaging with its promoter region. Subsequently, YTHDF1 associates with m6A‐modified ATG2A and ATG14 mRNA, thereby facilitating the translational processes of autophagy‐related genes ATG2A and ATG14. This intricate cascade amplifies the autophagic process and contributes significantly to the pathogenesis of autophagy‐associated malignancies.^115^

Recent investigations have illuminated that nicotinamide adenine dinucleotide phosphate (NADP) and NAD are also instrumental in modulating mRNA m6A modification. Specifically, NADP serves to potentiate the enzymatic activity of the RNA demethylase FTO, thereby fine‐tuning mRNA m6A methylation profiles, which subsequently wield an influence on adipocyte differentiation during the adipogenesis process. This insight delineates a direct nexus between cellular metabolic processes and the intricate regulatory mechanisms of RNA m6A methylation.^116^

Moreover, the accretion of lactate within the tumor microenvironment has been shown to instigate histone H3K18 lactylation, engendering elevated expression of Mettl3 and m6A modification of Jak1 mRNA within tumor‐infiltrating myeloid cells. This sequential cascade amplifies the translation of Jak1 mRNA and fosters the phosphorylation of STAT3, thereby facilitating immune‐suppressive functionalities in these myeloid cells and mediating mechanisms for tumor immune evasion.^117^ Furthermore, inhibiting glycolysis or lactate dehydrogenase (LDH) enzymes, specifically LDHA and LDHB, culminates in a lactate decrement, concomitantly attenuating H3K18 lactylation modifications that govern the transcription of the m6A reader protein YTHDF2.^118^ Notably, YTHDF2 possesses the ability to recognize m6A modifications on the 3′ UTR of tumor‐suppressor genes PER1 and TP53 mRNA, thereby diminishing their mRNA stability, particularly in ocular melanoma contexts.^118^

In addition to m6A modifications, other methylation variances are also subject to modulation by metabolic pathways or specific metabolites. For instance, upon the assimilation of fatty acids derived from omental adipocytes, gastric cancer cells experience an upregulation in the transcription factor E2F1. This elevation subsequently propels the expression of m5C methyltransferase NSUN2 via cis‐regulatory mechanisms.^119^ NSUN2, acting through m5C modifications, modulates the stability of ORAI2 mRNA, thereby facilitating its expression and, subsequently, promoting peritoneal metastasis in gastric cancer. In this complex regulatory landscape, YBX1 operates as a “reader,” recognizing and binding to the m5C modification site on ORAI2 and enhancing its mRNA stability.^119^

Currently, burgeoning empirical evidence is increasingly establishing a compelling correlation between dysregulated RNA methylation and perturbed tumor metabolism, thereby contributing to the complexity of tumor progression. The frontier of anticancer therapeutics is being reshaped by an emergent focus on developing drugs targeting RNA methylation (Table 1). Nonetheless, the intricate mechanisms that interlink RNA methylation dysregulation with tumor metabolism warrant more comprehensive, in‐depth investigation. Investigations aimed at drug development targeting RNA methylation‐modulating proteins are still nascent. Hence, the synthesis of more selective inhibitors or activators targeting these RNA methylation‐modulating entities holds the promise for furthering advancements in the burgeoning field of RNA‐based precision medicine.

**TABLE 1:** RNA m6A modification, targets, and drugs in clinical trials.

Target	Inhibitor	Mechanism	Cancer type	Clinical trial	Phase	Status	References
METTL3	STC‐15	Inhibiting the catalytic activity of METTL3	Acute myeloid leukemia	NCT05584111	I	Recruiting	N/A
	STM2457	Competitive binding to the binding site of the SAM	myeloid leukemia	N/A	N/A	N/A	^120^
FTO	Entacapone	Regulating the FTO–FOXO1 signaling pathway	Gastrointestinal stromal tumor	NCT04006769	I	Recruiting	^121^
	R‐2HG	Activating expression of RARA	Leukemia	N/A	N/A	N/A	^122^
	FB23‐2	Activating expression of ASB2	Leukemia	N/A	N/A	N/A	^123^
	Saikosaponin‐D	Increasing global m6A RNA methylation	Leukemia	N/A	N/A	N/A	^124^
	CS1/CS2	Inhibiting expression of immune checkpoint genes	Leukemia	N/A	N/A	N/A	^125^
	Dac51	Blocking immune evasion	Lung cancer and melanoma	N/A	N/A	N/A	^126^
ALKBH5	ALK‐04	Reducing m6A levels, abnormal splicing, Mct4/Slc16a3 expression, and lactate level	Melanoma	N/A	N/A	N/A	^127^
IGF2BP2/3	CWI1‐2	Inhibiting glutamine metabolism regulated by MYC, GPT2, and SLC1A5	Acute myeloid leukemia	N/A	N/A	N/A	^128^

##### Others

Phospholipids constitute a pivotal component of cellular membranes. While the primary understanding of phospholipids emanates from their salient biological functions within these membranes, it should be acknowledged that phospholipids and their derivatives, namely inositol phosphates (IPs), are ubiquitously present in the cellular nucleus and fulfill consequential biological roles therein.^129^

Phosphatidylethanolamine (PE) methylation serves as a paramount consumer of SAM. This methylation event participates in catalyzing the transsulfuration pathways imperative for the biosynthesis of cysteine and glutathione (GSH). Diminished PE methylation culminates in the intracellular accretion of SAM, thereby engendering hypermethylation of both histones and the key phosphatase PP2A. In the absence of PE methylation, particular loci on histones function as methyl sinks, catalyzing the conversion of SAM to SAH.^30^ Inositol pyrophosphates represent a distinct class of phospholipids characterized by a dynamically regulated metabolism orchestrated by inositol hexakisphosphate kinases (IP6Ks) and diphosphoinositol pentakisphosphate kinases (PPIP5K/VIP). Notably, Burton et al.^130^ unveiled that IP6K1 localizes to the nucleus where it interacts with Jumonji domain‐containing 2C (JMJD2C) HDM and modulates H3K9me3. Their in vitro analyses divulged that IP6K1 knockout instigated a decrease in H3K9me3 levels, counterbalanced by a surge in H3K9 acetylation and H3S10 phosphorylation, thereby elucidating the regulatory role of IP6K1 in specific histone modifications through JMJD2C inhibition.^130^

Phosphatidylinositol 4,5‐bisphosphate (PIP2), a derivative of inositol phosphoric acid, engages in complex interactions with UBF, RNA polymerase I, and prefibrillarin, thus modulating the transcriptional activity of RNA polymerase I and nucleolar RNA processing.^131^ In human osteosarcoma cells, PIP2 facilitates transcription by binding to the Ser5‐phosphorylated RNA polymerase II complex. Concurrently, Myosin Phosphatase Rho‐Interacting Protein mediates the recruitment of Tyr1‐phosphorylated CTD (Tyr1P‐CTD) to PIP2‐enriched nuclear structures, thereby modulating transcriptional elongation.^132^ Sphingosine 1‐phosphate (S1P), a vital component of bilayer membranes, exerts critical regulatory functions in cellular processes. Nuclear S1P interacts with HDAC1 and HDAC2, inhibiting their catalytic activity and thus modulating histone acetylation profiles.^133^ Elevated levels of nuclear S1P, as a result of sphingosine kinase upregulation, attenuate HDAC enzyme activities and augment p53 acetylation. IPs have been implicated in an array of nuclear functions such as DNA damage response, chromatin remodeling, mRNA export, and gene expression.^134^ IPs can potentiate HDAC activity by facilitating interactions between the catalytic domains and the SANT (Swi3, Ada2, nuclear receptor corepressor [N‐Cor], and TFIIIB) motif in almost all HDAC complexes, barring those that incorporate the Sin3 transcriptional corepressor factor.^135^

Collectively, these studies elucidate that aberrant nuclear lipid metabolism is intrinsically entangled with epigenomic regulation and may serve as a contributory factor in tumorigenesis. Despite the valuable insights garnered, our comprehension of nuclear lipid metabolism remains conspicuously incomplete. Additional research is imperative for elucidating these multifaceted molecular mechanisms, and may proffer innovative avenues for targeted cancer therapeutics.

#### 2.2.2 Nuclear function of metabolic enzymes

Emerging evidence posits that a multitude of metabolic enzymes, typically confined to the cytoplasm or mitochondria, can translocate into the nucleus and exercise regulatory influence over gene transcription by altering chromatin conformation. In addition to this epigenetic role, these enzymes execute additional nuclear functions that encompass gene transcription, DNA damage repair, and posttranslational modifications of proteins (Figure 2). These myriad functions collectively foster tumoral growth and pose impediments to effective cancer treatments. The “moonlighting” roles of these metabolic enzymes within the nuclear milieu are elaborated upon below.

##### Hexokinase 2

Recent investigations have elucidated that hexokinase 2 (HK2), an enzyme belonging to the transferase kinase category that phosphorylates glucose to glucose‐6‐phosphate, is translocated to the nucleus in direct association with the DNA‐binding transcriptional repressor Mig1. This translocation is modulated by glucose concentration and is under the regulatory influence of phosphorylation by Snf1 kinase and dephosphorylation by the Glc7‐Reg1 phosphatase complex.^136^, ^137^, ^138^ Subsequent studies indicate that nuclear HK2 acts as an activator for nuclear factor‐erythroid 2‐associated factor 2 (NRF2), a transcription factor that mitigates oxidative damage under conditions of metabolic stress in glioma cells.^139^ The AKT pathway is implicated in regulating nuclear accumulation of HK2, thereby augmenting its association with mitochondria and enhancing glucose uptake in cancer cells.^138^ In HeLa cells, AKT inhibitor IV (Ai4) serves to intensify the nuclear presence of HK2; conversely, in MDA‐MB‐231 breast cancer cells, Ai4 merely redistributes HK2 throughout the cytoplasm without inducing its nuclear accumulation.^140^ High‐glucose conditions retain glucokinase, a hexokinase isozyme, in the cytoplasm, whereas a glucose‐deficient environment instigates its nuclear translocation, a process reliant on its interaction with glucokinase regulatory protein.^141^, ^142^ Existing research posits that in both leukemic and standard hematopoietic stem cells, HK2 localization to the mitochondria and nucleus allows its interaction with nuclear proteins to regulate chromatin accessibility, thereby enhancing chromatin openness in leukemic stem cells^143^ (Figures 1 and 2).

Moreover, HK2 contains highly conserved cysteine residues that are sensitive to redox modification in response to reactive oxygen species (ROS) stimulation. For example, dehydroascorbic acid (DHA) forms a covalent bond with the active cysteine site of HK1, leading to irreversible enzymatic inactivity.^144^, ^145^

Benitrobenrazide, an innovative selective HK2 inhibitor, demonstrates the capability to target the binding site and preclude glucose association with HK2, thus efficaciously inhibiting pancreatic cancer growth by disrupting glycolytic pathways.^146^ Nevertheless, its potential impact on nuclear HK2 remains ambiguous. 3‐Bromopyruvic acid, a pyruvate analogue, is another plausible HK2 inhibitor,^147^ which has been shown to induce apoptosis and inhibit proliferation in a range of cancer types.^147^ Prior research has validated that lonidamine (LND), an indazole‐3‐carboxylic acid derivative, specifically inhibits the aerobic glycolysis and energy metabolism of cancer cells.^148^, ^149^ Additionally, LND fosters the generation of ROS and compromises melanoma cell viability by inhibiting the succinate panquinone reductase activity of respiratory complex II^150^ (Table 2).

**TABLE 2:** Nuclear metabolic enzymes, their roles, and drugs in cancer.

Metabolic enzyme	Nuclear function	Mechanism	Cancer type	Drugs	Chemical structure	Clinical trial	References
HK2	Provide protection against oxidative stress	Activate NRF 2	Glioma	Benitrobenrazide	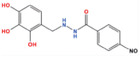	Preclinical	^146^
	Regulate chromatin openness	Interact with nuclear proteins	Acute myeloid leukemia	3‐Bromopyruvic acid	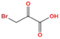	Preclinical	^147^
				Lonidamine	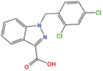	N/A	^149^, ^150^
PFK1	Activate CXCR4 and T‐ALL cells invasion	Interacts with c‐Myc	Acute lymphoblastic leukemia	Citrate	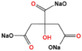	Preclinical	^155^, ^156^
				ML251	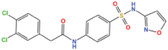	N/A	^157^
GAPDH	Induce nuclear protein degradation and cell apoptosis	GAPDH S‐nitrosylation enhances its interaction with and stabilization of Siah1	Neuroblastoma/ prostate cancer	Selegiline	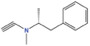	NCT04586543 (Phase I)	^158^, ^165^, ^166^
	Increase expression of histone H2B	AMPK‐induced phosphorylation of GAPDH interacts with Oct‐1 and OCA‐S	Osteosarcoma/cervical cancer	Heptelidic acid	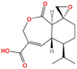	Preclinical	^167^
	Protection and maintenance of telomeres	Interacts with telomere DNA	Lung cancer	1,2,3,4,6‐penta‐O‐galloyl‐β‐d‐glucopyranose	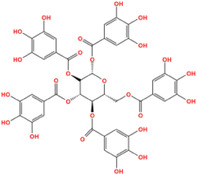	Preclinical	^168^
PFKFB4	Stimulate gene expression of metabolic enzymes and tumor growth	Phosphorylate SRC‐3	Breast cancer	5‐(N‐(8‐methoxy‐4‐quinolyl)amino)pentylnitrate	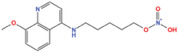	Preclinical	^171^
PKM2	Transcription	Phosphorylate STAT3 and H3	Glioma/retinoblastoma	Shikonin	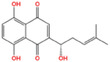	Preclinical	^184^, ^185^, ^186^
	Cell cycle progression/proliferation	Phosphorylate H3	Retinoblastoma/glioma	Alkannin	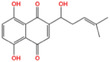	Preclinical	^186^
	Upregulation of glycolytic enzymes	Promote HIF‐binding activity to DNA	Cervical cancer	Scutellarin	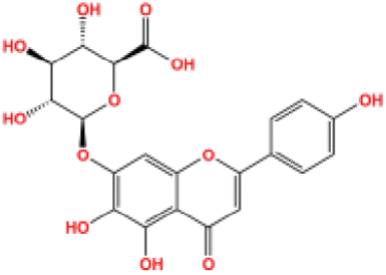	Preclinical	^187^
LDH	Histone H3 hyper‐acetylation/damage response genes	Increase nuclear acetyl‐CoA and lactate	Hepatocellular carcinoma	Galloflavin	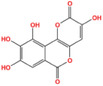	Preclinical	^190^, ^191^
	NRF2 antioxidant genes and Wnt target genes/histone H3K79 hypermethylation	Gains a noncanonical enzyme activity to produce α‐hydroxybutyrate and triggers DOT1L	Cervical cancer	Oxamic acid sodium	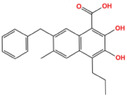	N/A	^192^
				FX11	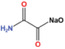	Preclinical	^193^, ^194^
Fumarase	DNA damage and repair	Preserve histone H3 methylation at double‐strand break (DSB) regions	Hepatocellular carcinoma	Fumarate hydratase‐IN‐1	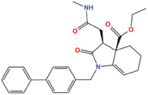	Preclinical	^198^
	Transcription	Interact with AF2 at promoter region	Pancreatic cancer				
	Inhibit cell growth	Inhibit lysine‐specific demethylase 2A (KDM2A) and promotion of histone H3K36me2	Pancreatic cancer				
FBP	Inhibit HIF nuclear function	Interact with HIF inhibitory domain and restrain the nuclear function of HIF1α and HIF2α	Renal carcinoma	FBPase‐1 inhibitor‐1	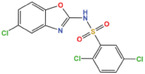	Preclinical	^201^
	Suppress mitochondrial respiration and the TCA cycle in a c‐Myc‐dependent manner	Inhibit NRF1 and TFAM‐dependent mitochondrial biogenesis gene expression	Sarcoma	FBPase‐IN‐1	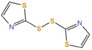	Preclinical	^202^

##### Phosphofructokinase 1

Within the glycolytic cascade, phosphofructokinase 1 (PFK1) functions as a rate‐limiting enzyme, facilitating the conversion of fructose 6‐phosphate to fructose 1,6‐bisphosphate. Various tetrameric isozymic variants of PFK1 coalesce to produce three distinct subunit types—namely, PFKM (muscle), PFKL (liver), and PFKP (platelet).^151^, ^152^, ^153^ PFKP, the predominant isoform of PFK1, is discernible in T cell acute lymphoblastic leukemia (T‐ALL). D3/cyclin‐dependent kinase 6 (CDK6) phosphorylates PFKP at the Ser 679 site, thereby promoting an interaction with importin 9 and culminating in nuclear translocation of PFKP.^154^ The chemokine receptor, C‐X‐C chemokine receptor type 4 (CXCR4), modulates the homing and infiltration of human leukemia cells, consequently facilitating T‐ALL cell invasion. Nuclear PFKP elevates CXCR4 expression via interaction with c‐Myc^154^ (Figures 1 and 2). Citrate, an efficacious physiological inhibitor of PFK1, has been shown to significantly attenuate aerobic glycolysis and tumor proliferation in non‐small cell lung cancer.^155^, ^156^ ML251, another potent PFK inhibitor, exhibits promise, although its precise role in oncological therapy remains uncertain^157^ (Table 2).

##### Glyceraldehyde‐3‐phosphate dehydrogenase

Glyceraldehyde‐3‐phosphate dehydrogenase (GAPDH) plays an instrumental role in the glycolytic pathway, catalyzing the conversion of glyceraldehyde 3‐phosphate to 1,3‐bisphosphoglycerate. Remarkably, it also exhibits a proclivity for direct interaction with the substrates of the G1/S‐specific cyclin E‐CDK2 complex, specifically OCT1 and NPAT, thereby orchestrating the transcriptional regulation of histone genes (Figures 1 and 2).

The enzyme undergoes posttranslational modification via S‐nitrosylation at the Cys150 site, a process incited by the release of nitric oxide (NO) during the initiation of apoptosis. This modification facilitates GAPDH's binding to Siah1, an E3 ubiquitin ligase, and culminates in its nuclear translocation, ultimately inducing cellular apoptosis. Importantly, S‐nitrosylation accentuates GAPDH's interaction with Siah1, whose nuclear localization signal acts as a catalyst for GAPDH's nuclear translocation.^158^ Within the nuclear domain, GAPDH stabilizes Siah1, leading to ubiquitination and subsequent degradation of specific nuclear proteins such as the N‐CoR, thereby triggering neuronal death.^158^

In glucose‐deprived conditions, cytoplasmic GAPDH is phosphorylated at the Ser122 site by activated AMPK. This phosphorylation serves as an impetus for GAPDH's nuclear translocation. Once localized to the nucleus, GAPDH engages in direct interactions with Sirt1, effectively displacing its repressor and activating the enzyme.^159^ Furthermore, nuclear GAPDH enhances the expression levels of histone H2B during the S‐phase, achieved through the assembly of a complex with Oct‐1 and OCA‐S (Oct‐1 coactivator at the S phase).^160^

Ceramide exerts a regulatory influence over GAPDH's nuclear localization in lung cancer cells through a cell cycle‐dependent mechanism, in addition to inhibiting its telomere‐binding function. Intriguingly, a rapid telomeric shortening is observed upon the inhibition of GAPDH expression, highlighting the critical role that nuclear GAPDH plays in telomere maintenance and protection.^161^

Under conditions of oxidative stress, cysteine 152 (Cys152), a conserved active site within GAPDH, undergoes posttranslational modifications via sulfonation or glutathionylation. Such redox‐mediated modifications possess the capacity to attenuate GAPDH enzymatic activity, thereby rerouting metabolic flux away from glycolysis toward the pentose phosphate pathway (PPP).^162^ This metabolic diversion serves to augment the viability and adaptive capabilities of neoplastic cells. Intriguingly, the oxidative alteration of Cys152 is not directly orchestrated by ROS; rather, it is facilitated through an intermediary cysteine at the 156 position (Cys156), which undergoes initial oxidation followed by a proton relay mechanism to Cys152.^162^, ^163^

High‐dose vitamin C therapy proves efficacious in mitigating the extensive depletion of GSH as well as the accumulation of ROS, thereby expediting the glutathionylation of GAPDH at the Cys152 site. Such modulation effectively inactivates GAPDH and exerts inhibitory effects on colon cancer cells harboring KRAS and BRAF mutations, primarily through the obstruction of glycolytic pathways.^164^ Nonetheless, it is imperative to acknowledge that the ROS accumulation instigated by vitamin C is a nonspecific phenomenon; therefore, the potential for oxidative modifications to proteins other than GAPDH cannot be categorically dismissed, thereby implicating a more generalized tumor‐suppressive effect.

In a therapeutic context, selegiline—a pharmacological agent employed in Parkinson's disease treatment—can inhibit the S‐nitrosylation of GAPDH, thereby precluding its interaction with Siah1 and constraining the nuclear translocation of the GAPDH‐Siah complex, thus limiting cellular apoptosis.^158^, ^165^, ^166^ Heptelidic acid, also known as Koningic acid, serves as a specialized GAPDH inhibitor, attenuating Etoposide‐induced apoptosis through the downregulation of caspases.^167^ 1,2,3,4,6‐penta‐O‐galloyl‐β‐d‐glucopyranose has been reported to compete with NAD+ and inorganic phosphate (Pi) to impede GAPDH activity; however, its inhibitory efficacy in tumor models remains to be empirically validated^168^ (Table 2). In summation, GAPDH inhibitors hold promising therapeutic potential for cancer treatment applications.

##### N6‐phosphofructo‐2‐kinase/fructose‐2,6‐bisphos‐phatase 4

N6‐phosphofructo‐2‐kinase/fructose‐2,6‐bisphos‐phatase 4 (PFKFB4) serves as an indispensable catalyst in the glycolytic pathway and is manifestly overexpressed in neoplastic cells^169^ (Figures 1 and 2). Emerging evidence delineates the modulatory impact of PFKFB4 on the biological transcriptional machinery, specifically through its regulatory interaction with the oncogenic steroid receptor coactivator‐3 (SRC‐3). SRC‐3, in turn, liaises with a multitude of nuclear receptors. A seminal study by Dasgupta et al.^170^ illuminates that tumors positive for ERs exhibit concomitantly elevated levels of PFKFB4 and phosphorylated SRC‐3, thereby indicating a correlative association between the two entities.^171^

SRC‐3 undergoes specific phosphorylation at the serine 857 residue, mediated by the protein kinase activity of PFKFB4. Once phosphorylated, SRC‐3 engages in intricate interactions with ATF4 at the gene promoter locales, thereby engendering an upsurge in the expression of an array of metabolic enzymes. Noteworthy among these are transketolase, xanthine dehydrogenase, and adenosine monophosphate deaminase‐1.^170^ Importantly, targeted inhibition of either SRC‐3 or PFKFB4 culminates in the attenuation of breast tumor development in murine models and curtails metastatic propagation to pulmonary tissues when assessed in an orthotopic milieu. These salient findings accentuate the pivotal role of PFKFB4, not merely as a glycolytic enzyme but as a protein kinase, in orchestrating the transcriptional landscape through the direct phosphorylation of the coactivator SRC‐3.^170^, ^171^

In the realm of therapeutic interventions, 5‐(N‐(8‐methoxy‐4‐quinolyl)amino)pentyl nitrate (5MPN) has been unequivocally identified as a potent, orally bioavailable, and selective inhibitor of PFKFB4. 5MPN operates as a competitive antagonist at the F6P binding site, consequently exerting a targeted inhibition on glucose metabolism. This inhibition effectually constrains the proliferative trajectory of a diverse range of malignancies^171^ (Table 2).

##### Pyruvate kinase

Pyruvate kinase (PK) serves as a pivotal enzyme in the glycolytic pathway, facilitating the conversion of phosphoenolpyruvate (PEP) and adenosine diphosphate into pyruvate and ATP.^172^ Notably, two isoforms of PK, namely PKM1 and PKM2, have been delineated, with the latter uniquely localized in the nuclear compartment.^173^ Within the nucleus, PKM2 predominantly exists in either dimeric or monomeric configurations, whereas its cytosolic analogue principally manifests as a tetramer.^174^

In addition to its canonical role in glycolysis, PKM2 exhibits multifunctional capabilities as a protein kinase. In the nuclear milieu, PKM2 dimers function as active protein kinases, whereas the tetrameric form operates as an efficacious PK.^175^ Employing PEP as the phosphoryl donor, nuclear PKM2 is implicated in the phosphorylation of STAT3 and histone H3, thereby modulating gene transcription^176^, ^177^, ^178^ (Figures 1 and 2). In oncogenic contexts, nuclear PKM2 specifically phosphorylates STAT3 at the 705th amino acid residue, thereby functioning as a transcriptional coactivator to upregulate MEK5 (also known as MAP2K5) expression.^176^ Furthermore, nuclear PKM2 and STAT3 synergize, enabling the facilitation of STAT3 phosphorylation and concomitantly promoting Th17 cell differentiation; however, PKM2 is dispensable for metabolic reprogramming or proliferation in Th17 cells.^177^

In response to epidermal growth factor (EGF) stimuli, PKM2 directly phosphorylates and associates with histone H3. This interaction is requisite for K9 histone H3 acetylation, and such PKM2‐dependent chromatin modifications bear significant implications for brain carcinogenesis, tumor cell proliferation, cell cycle dynamics, and EGF‐mediated expression of cyclin D1 and c‐Myc.^176^ In retinoblastoma, hepatocyte growth factor‐induced CMET‐dependent signaling leads to the phosphorylation of ERK 1/2, subsequently enhancing the nuclear translocation of PKM2. Therein, PKM2 collaborates with histone H3, thereby stimulating tumor cell growth and facilitating C‐MET‐dependent synthesis of cyclin D1 and c‐Myc.^178^

Furthermore, PKM2 modulates the G1/S phase transition by orchestrating the expression of cyclin D1 and effecting phosphorylation of the Bub3 constituent of the spindle assembly checkpoint complex (SAC). Intriguingly, Jiang et al. reported that glioblastoma prognosis correlates with the phosphorylation levels of Bub3 at the Y207 residue.^179^ PKM2 also functions as a cotranscriptional modulator, independent of its kinase activity, thereby bolstering HIF binding to DNA. This interaction initiates a positive feedback loop with HIF‐1, culminating in the upregulation of multiple glycolytic enzymes, inclusive of PKM2 itself.^180^, ^181^

The functionality of nuclear PKM2 extends beyond enzymatic catalysis to the regulation of gene expression, chromatin modification, and cell cycle progression, thereby implicating it in oncogenesis. Nevertheless, comprehensive investigations remain imperative for elucidating the intricate roles of PKM2 as a protein kinase.

Emerging research posits that ROS exert a direct modulatory impact on PKM2, subsequently altering tumor metabolic dynamics. Under conditions of oxidative stress, the acetylation state of PKM2 diminishes, thereby resisting lysosomal degradation and contributing to pharmacological resistance in renal cell carcinoma.^182^ Additional studies have reported that PKM2 undergoes oxidation and consequent inactivation at the cysteine 358 residue (Cys358). This modification impedes glycolytic flux and redirects cellular metabolism toward the PPP, thereby generating NADPH essential for ROS detoxification. This metabolic shift confers a survival advantage to lung cancer cells under oxidative stress.^183^

Phytochemicals such as Shikonin and alkannin, derived from traditional Chinese herbal medicine, have been identified as specific PKM2 inhibitors.^184^, ^185^, ^186^ Historically employed for the treatment of dermatitis, burns, and traumatic injuries,^182^ recent investigations have unveiled Shikonin's antitumorigenic properties via PKM2 inhibition, resulting in the suppression of glycolytic activity.^184^, ^185^, ^186^ Another PKM2 antagonist, Scutellarin, exhibits the ability to directly bind to PKM2 and attenuate its enzymatic function. Subsequently, this interaction diminishes glycolytic metabolism and heightens nuclear translocation of PKM2, thereby mitigating apoptotic pathways in tumor cells through the modulation of the MEK/ERK/PIN1 signaling axis^187^ (Table 2).

##### Lactate dehydrogenase

LDH is a pivotal enzyme that mediates the interconversion between pyruvate and lactate, while concurrently catalyzing the redox reaction involving NADH and NAD^+^ ^188^ (Figures 1 and 2). As hepatic function deteriorates, LDH accrues within the nuclear compartment, leading to elevated levels of acetyl‐CoA and lactate, as well as the induction of histone H3 hyper‐acetylation and damage response genes.^188^ Interestingly, an aggregation of intracellular ROS can disassemble the LDHA tetramer, concurrently augmenting nuclear LDHA concentration in Human papilloma virus (HPV) 16 E7 oncoprotein‐positive cervical cancer cells.^189^ In a noncanonical enzymatic function, nuclear LDHA catalyzes the production of α‐hydroxybutyrate, which facilitates histone H3K79 hypermethylation via methyltransferase disruptor of telomeric silencing 1‐like (DOT1L). This modification upregulates target genes in the Wnt signaling pathway and NRF2 antioxidant genes, thereby implicating it in oncogenesis.^189^

Therapeutically, Galloflavin has been identified as a putative LDH inhibitor that induces cancer cell apoptosis by obstructing glycolysis and ATP synthesis.^190^, ^191^ Oxamate, a classic LDHA inhibitor, operates by competitively inhibiting the enzyme's affinity for its substrate, pyruvate.^192^ Prior studies substantiate Oxamate's potential to abrogate proliferation, invasion, and migration across a plethora of cancers, including but not limited to breast, liver, and non‐small cell lung cancer, whilst simultaneously enhancing the efficacy of other antineoplastic agents and radiotherapy.^192^ Notwithstanding, the inhibitory effect of oxalate on LDHA is characterized as nonspecific and relatively weak. Conversely, FX‐11 has garnered attention as a potent, selective, and competitive inhibitor specific to LDHA, significantly reducing ATP levels while inducing ROS generation and consequent tumor cell death^193^, ^194^ (Table 2).

##### Fumarase

Within the TCA (tricarboxylic acid) cycle, fumarase, also known as FH, serves as an enzymatic catalyst facilitating the reversible hydration and dehydration of fumarate to malate, thereby promoting energy production via the generation of reduced NAD (NADH) (Figures 1 and 2). Remarkably, in response to genotoxic stress, fumarase catalyzes the transformation of fumarate into malate, a metabolite that can be translocated to the nuclear compartment and participate in DNA repair mechanisms.^195^ Deficiency in fumarase functionality can drive oncogenesis, primarily by compromising DNA repair processes and consequently facilitating the accrual of genomic mutations. Intriguingly, localized fumarate production within subnuclear domains preserves histone H3 methylation at sites of DSBs, representing a critical component of the cellular DNA damage response architecture.^195^, ^196^ During glucose scarcity, AMPK phosphorylates fumarase, which then interacts with the transcription factor ATF2. This complex translocates to the promoters of ATF2‐regulated genes, where locally generated fumarate inhibits the activity of KG‐dependent demethylase (KDM2A), culminating in dimethylation of H3K36 and ensuing cell growth arrest.^197^ Notably, FH‐IN‐1, a cell‐permeable fumarase inhibitor, has displayed antineoplastic activity across a diverse array of malignancies^198^ (Table 2).

##### Fructose‐1,6‐bisphosphatase

FBP acts as a rate‐limiting enzyme, orchestrating the hydrolysis of fructose 1,6‐bisphosphate to fructose 6‐phosphate, a pivotal step in gluconeogenesis — the biochemical antithesis of glycolysis. Vertebrates express two isozymic forms of FBP: FBP1, predominantly expressed in hepatic and renal tissues, and FBP2, which is primarily localized in skeletal muscle and mesenchymal tissues.^199^ Recent investigations have elucidated that FBP1 can translocate to the nucleus, where it directly engages with the HIF “inhibitory domain,” thus attenuating the activities of HIF1α and HIF2α (also referred to as EPAS1) (Figures 1 and 2). Such downregulation of FBP1 in renal carcinoma cells abolishes its HIF inhibitory function, thereby exacerbating tumorigenic processes.^200^ Complementary research in sarcoma has ascertained that FBP2 is epigenetically silenced across various sarcoma subtypes and its presence inhibits cellular proliferation. Notably, nuclear FBP2 suppresses the expression of mitochondrial biogenesis genes regulated by nuclear respiratory factor 1 (NRF1) and transcription factor A (TFAM). This results in diminished mitochondrial respiration and TCA cycle activity in a c‐Myc‐dependent manner. Specifically, c‐Myc colocalizes with nuclear FBP2 at the TFAM binding site, leading to the inhibition of TFAM expression and, consequently, sarcoma tumorigenesis.^199^ With regard to therapeutic avenues, FBPase‐1 inhibitor‐1 functions as an allosteric inhibitor of FBP,^201^ while FBPase‐IN‐1 has been identified as another FBP inhibitor with the capability to ameliorate hyperglycemia and glucose intolerance in Type 2 diabetes.^202^ However, the efficacy of these inhibitors in the context of cancer remains unexplored (Table 2).

## 3 THERAPEUTIC STRATEGIES AND CLINICAL APPLICATIONS TO TARGET NUCLEAR METABOLIC REGULATION

Since the seminal discovery of metabolic aberrations in neoplastic tissues, the field of cancer metabolism has witnessed significant advancements over the past several decades. Emerging therapeutic paradigms grounded in metabolic regulation have been developed and integrated into the clinical management of malignancies. These include the employment of small‐molecule inhibitors targeting pivotal metabolic pathways, the combination of metabolic interventions with immune checkpoint inhibitors, and multimodality treatment strategies that incorporate metabolic drugs into conventional oncological regimens such as surgical resection, radiation, and chemotherapy.^8^, ^9^, ^11^, ^203^ Nonetheless, the therapeutic landscape focusing explicitly on nuclear metabolic signaling remains comparatively nascent, despite its critical implications in cancer metabolism. In the subsequent sections, we delineate the extant nuclear metabolism‐centric therapeutic strategies and their clinical ramifications, and provide insights for the potential translation or alternative approach of tumor metabolism‐based preclinical and clinical study.

### 3.1 Targeting nucleotide metabolism, RNA, and DNA synthesis

In the clinical armamentarium against cancer, metabolic chemotherapy agents are predominantly classified into two categories: folate antagonists and nucleotide base analogs. These compounds primarily exert their antineoplastic effects by obstructing nucleotide synthesis and thereby impeding cellular replication.^204^ Folate antagonists, represented by drugs such as methotrexate and pemetrexed, inhibit dihydrofolate reductase, effectively thwarting the conversion of dihydrofolate into its physiologically active form, THF. This enzymatic blockade disrupts the one‐carbon unit transfer essential for purine and pyrimidine nucleotide biosynthesis, thereby stalling DNA synthesis.^205^, ^206^, ^207^ Base analogs like 5‐fluorouracil, gemcitabine, thioguanine, and fludarabine operate by mimicking nucleotide bases, thereby disrupting the integrity of DNA synthesis.^204^, ^207^, ^208^ As such, the therapeutic modulation of nucleotide metabolism presents an efficacious avenue in oncological treatment.

### 3.2 Targeting nuclear metabolic products via inhibiting metabolic pathway

As elucidated in preceding discussions, key metabolic pathways such as the TCA cycle, one‐carbon metabolism, and glucose metabolism are instrumental in regulating nuclear processes including RNA modifications, DNA methylation, and histone modifications. These pathways supply essential metabolites that function as cofactors in these nuclear modifications. Additionally, the intermediates generated from these metabolic cycles contribute to nucleotide synthesis, ribose generation, and the production of purine and pyrimidine nucleotides.^202^ The strategic attenuation or abrogation of these substrate supplies by targeting key metabolic pathways could unlock novel therapeutic avenues in the battle against cancer. However, the realization of these promising therapies is contingent upon the development of highly specific and effective inhibitors.

### 3.3 Targeting nuclear metabolic enzymes

In addition to metabolites that wield significant epigenetic influence on oncogenesis, metabolic enzymes serve as pivotal regulatory entities in the trajectory of tumor development. Evidence suggests that these enzymes undergo nuclear translocation, where they manifest kinase or nonkinase functions that modulate gene transcription, DNA damage repair, and posttranslational modifications, thereby influencing oncogene expression (Table 2). Although pharmacological inhibitors targeting these nuclear metabolic enzymes have been synthesized, the extent to which these inhibitors modulate their intranuclear biological activities warrants further rigorous investigation. Continued research could elucidate the complex interrelationships between metabolic enzymes and nuclear processes, thus unveiling innovative paradigms for therapeutic interventions specifically aimed at nuclear metabolic regulation in neoplastic conditions.

### 3.4 Targeting metabolism‐related epigenetic modifications

The advancement of scientific research has laid bare a myriad of nuclear protein, DNA, and RNA modifications that serve as cardinal regulators of nuclear function and the transmission of epigenetic information. HDAC inhibitors, including compounds such as Vorinostat (Zolinza), Belinostat, Panobinostat (Faridak), and Romidepsin (Istodax), have received approval from the United States Food and Drug Administration (US FDA) and have demonstrated clinical efficacy in oncological applications^209^, ^210^ (Table 3). More recently, the histone methyltransferase inhibitor Tazemetostat achieved US FDA endorsement, thereby augmenting the available repertoire of therapeutic agents. Moreover, two DNMT inhibitors, 5‐azacytidine (Vidaza) and SGI‐110 (guadecitabine), have been recognized by both the US FDA and the European Medicines Agency (EMA), offering innovative therapeutic modalities for malignancy management. Concurrently, several prospective inhibitors targeting these epigenetic modifications are at various stages of clinical evaluation, portending future therapeutic advancements (Table 3). This burgeoning domain of research has catalyzed significant innovation within the realm of pharmacotherapy, fostering the development of agents with a high degree of specificity for these critical epigenetic modifications.

**TABLE 3:** Epigenetic modification, targets, and drugs in cancer.

Targets	Drugs	Mechanism	Cancer type	Clinical trial	Phase	Status	References
DNMT	5‐Azacytidine (Vidaza)	Reversing hypermethylation to restore transcription of tumor suppressor genes	Squamous cell carcinoma, head and neck cancer	NCT05317000	I	Recruiting	^221^
	SGI‐110 (guadecitabine)	The precursor drug of decitabine/ extending drug exposure	Leukemia	NCT02907359	III	Completed	^210^
	Decitabine (Dacogen)	Inhibit DNA methylation of tumor suppressor genes	Pancreatic cancer	NCT05360264	II	Recruiting	^222^
HDAC	Vorinostat (Zolinza)	Modulating chromatin conformation, inducing cellular differentiation, and blocking cell cycle	Rhabdomyosarcom	NCT04308330	I	Recruiting	^223^
	Belinostat	Inducing accumulation of acetylated histones and apoptosis‐related proteins	Refractory peripheral T‐cell lymphoma	NCT05170334	II	Recruiting	^224^
	Citarinostat	Decreasing Th2 cytokine production (i.e., IL‐4, IL‐5, IL‐6, IL‐10, and IL‐13)	Smoldering multiple myeloma	NCT02886065	I	Recruiting	^225^
	Entinostat	Promoting immune editing of tumor neoantigens and remodeling the tumor immune microenvironment	Breast cancer	NCT02115282	III	Recruiting	^226^
	Valproic acid	Inducing proteasomal degradation of HDAC2	Non‐small‐lung cancer	NCT01203735	II	N/A	^227^
	Trichostatin A	Inhibiting dendritic cell maturation andadipogenesis	Hematologic malignancies	NCT03838926	I	N/A	^228^, ^229^
	Tacedinaline (CI‐994)	Suppressing NF‐κB pathway	Lung cancer	NCT00005093	III	Completed	^230^
	Panobinostat (Faridak)	Modulating chromatin conformation, facilitating transcription, and inhibiting protein metabolism	Multiple myeloma	N/A	N/A	Approved (US FDA)	^231^
	Romidepsin (Istodax)	Inducing cell death	Cutaneous T‐cell lymphoma	N/A	N/A	Approved (US FDA)	^232^
	Chidamide	Inducing growth arrest and apoptosis and enhancing antitumor immunity	Peripheral T‐cell lymphoma	N/A	N/A	N/A	^233^
HMTs	Tazemetostat	Competitively inhibiting the methylation of histone lysine	Relapsed or refractory follicular lymphoma	NCT01897571	II	Completed	^234^
	Pinometostat	Reducing H3K79 methylation	Acute myeloid leukemia	NCT03701295	II	Completed	^235^
	GSK126	Suppressing antitumor immunity by driving production of myeloid‐derived suppressor cells	Leukemia	N/A	N/A	N/A	^236^
	AZ505	Inhibiting the binding of SMYD2 to miR‐125b promoter	Renal cell carcinoma	N/A	N/A	N/A	^237^
	EPZ015666	Inhibiting EMT by promoting expression of TET1 and increasing 5hmC	Cervical cancer	N/A	N/A	N/A	^238^
	MS023	Activation of interferon responses	Breast cancer	N/A	N/A	N/A	^239^
HDMs	GSK2879552	Inducing the expression of KDM1A‐targeted genes to counteract cell proliferation	Leukemia	NCT02177812	I	Terminated	^240^
	Seclidemstat (SP‐2577)	Suppressing the viability and metabolism of NK cells	Chronic myelomonocytic leukemia	NCT04734990	II	Recruiting	^241^
	JIB‐04	Inducing cell apoptosis	Liver cancer	N/A	N/A	N/A	^242^
	GSK‐J4	Inhibiting the JMJD3/UTX enzyme to upregulate H3K27me3 level	Acute myeloid leukemia	N/A	N/A	N/A	^243^
	ZY0511	Inducing H3K4 methylation at the promoter of GADD45B	Hepatocellular carcinoma	N/A	N/A	N/A	^244^

Abbreviations: DNMT, DNA methyltransferases; HDAC, histone deacetylases; HMTs, histone methylases; HDMs, histone demethylases.

### 3.5 Targeting RNA modifications

In contrast to the more mature understanding, we possess concerning protein and DNA modifications, the scientific comprehension of RNA modifications remains in its formative stage. This nascent understanding has led to ongoing research endeavors that have yet to fully elucidate the complexities inherent in RNA modifications. As a result, pharmacological inhibitors targeting these modifications are still in their incipient phases and have generally not progressed to the stage of clinical trials (Table 1). Nevertheless, the continuous, incremental advancements in this burgeoning field are propitious for the eventual identification and development of novel inhibitors capable of modulating RNA modifications, thereby enriching the compendium of therapeutic avenues available for future clinical applications.

### 3.6 Others

Beyond the aforementioned therapeutic strategies, alternative approaches are under exploration in both preclinical and clinical settings. These include: (1) manipulating transcription factor regulation as a means of controlling or responding to metabolic aberrations, such as the role played by Sterol Regulatory Element‐Binding Proteins (SREBPs) in lipid metabolism, and transcription factors like p53 and c‐Myc.^11^ (2) The synergistic application of nuclear metabolic inhibitors in conjunction with traditional therapeutic modalities, including surgical interventions,^211^, ^212^ radiotherapy,^213^, ^214^ chemotherapy,^215^, ^216^ and immunotherapy.^217^, ^218^ Such metabolic perturbations within cancer cells have been demonstrated to enhance their sensitivity to these conventional treatments. For instance, in reaction to radiation or chemotherapy, cancer cells invoke adaptive metabolic shifts that fortify mechanisms of radioresistance and chemoresistance.^219^ Interventions timed to disrupt this metabolic homeostasis may potentiate enhanced clinical responses. (3) The multifaceted targeting of multiple metabolic pathways. Given the metabolic heterogeneity or metabolic plasticity observed within tumors, monotherapeutic approaches targeting a single pathway often prove inadequate. Hence, polypharmacological strategies employing multiple inhibitors are being pursued for comprehensive cancer treatment.^220^ Despite these efforts, methodologies to specifically target nuclear metabolic signaling require further developmental refinement.

## 4 CONCLUSION AND PERSPECTIVES

**FIGURE 4:**
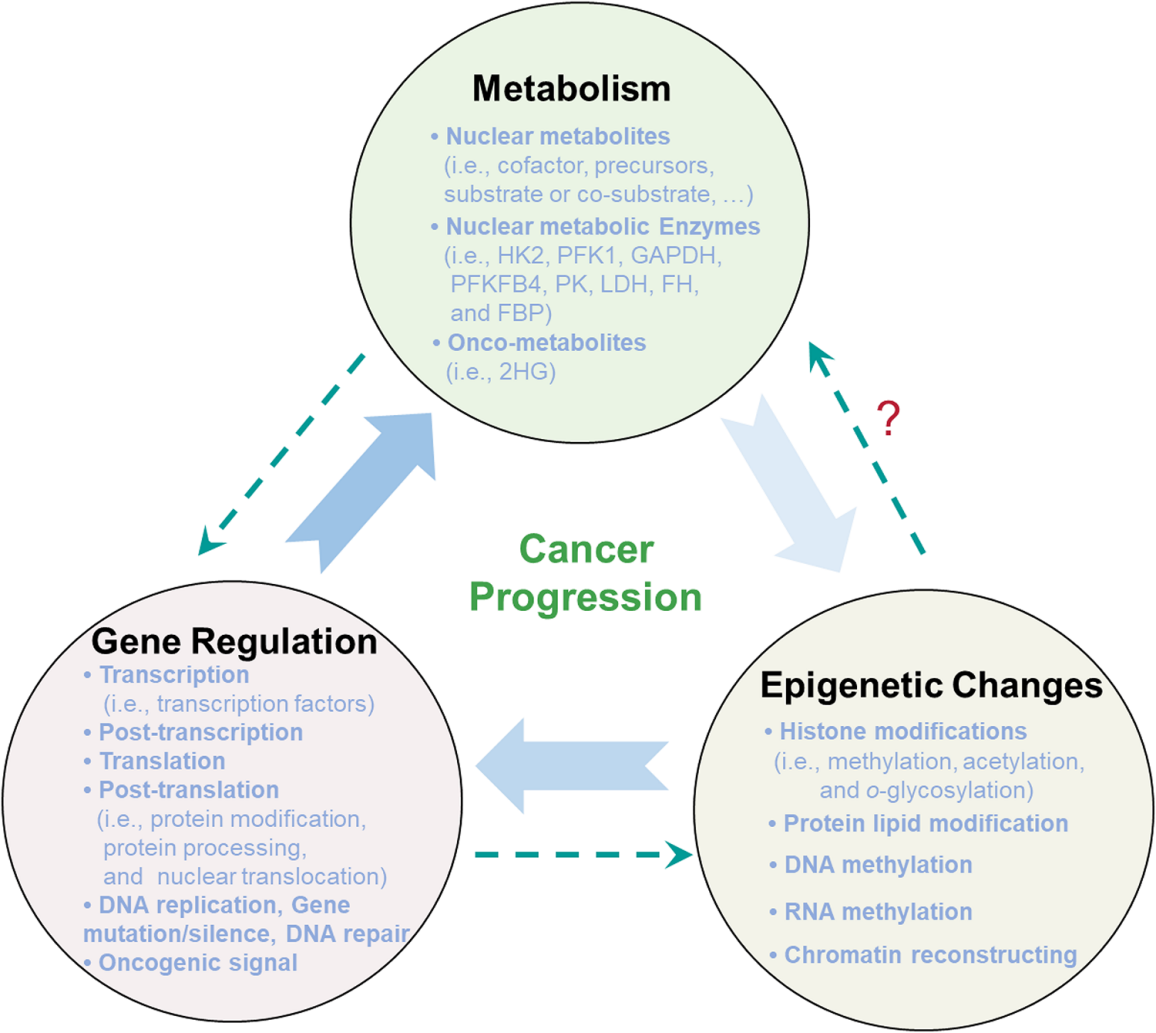
The positive feedback loop of metabolism–epigenetics–gene regulation. As delineated throughout this review, a positive feedback loop underpins the progression of malignant growth. Nuclear metabolites, which may be derived from extracellular sources, along with intranuclear metabolic enzymes, serve as cosubstrates, cofactors, or regulators in instigating epigenetic changes. These changes either promote or inhibit the expression of a myriad of genes, subsequently amplifying metabolic activity within the cell. Metabolism, in turn, exerts a direct influence on gene functionality via posttranslational modifications, protein processing, and nuclear translocation, among other mechanisms. Research exploring the direct ramifications of epigenetic changes on metabolic activity—specifically, how chromatin modifications may influence cellular metabolism via nongenomic routes—is relatively scant, underscoring the complexity of the interactions shaping malignant tumorigenesis.

Cellular metabolism and genomic regulation are inextricably intertwined, operating in a highly coordinated manner to facilitate rapid cellular adaptation to fluctuating extracellular nutrient conditions (Figure 4). A failure in this intricate regulatory balance can precipitate a cascade of adverse outcomes, including developmental aberrations, cellular apoptosis, and the onset of multifaceted pathologies. In navigating the complexities of a dynamic milieu—replete with fluctuating nutrient levels, growth factors, cytokines, and varying oxygen concentrations—metabolic enzymes undergo intricate regulatory modulations to satisfy the metabolic imperatives of tumorigenic growth. These modulations manifest through alterations in enzymatic activity, expression levels, and subcellular compartmentalization.

As elucidated in this review, a salient feedback loop involving metabolism, epigenetics, and gene regulation serves as a linchpin in the progression of malignancies (Figure 4). Within the tumor cell milieu, nuclear metabolites and metabolic enzymes—primarily of cytoplasmic origin—act as cosubstrates, cofactors, or regulators that influence epigenetic modifications of DNA and histones. These epigenetic alterations, in turn, modulate the expression profiles of relevant genes, thereby facilitating or amplifying the neoplastic metabolic landscape. Within the nuclear compartment, these entities execute their regulatory functions through several intricate mechanisms. Firstly, they modulate carcinoma‐associated gene expression primarily by influencing DNA and histone modifications as well as gene transcription. Metabolic byproducts, such as acyl‐CoA, SAM, fumarate, amino acids, α‐KG, 2‐HG, and NAD^+^, serve as requisite precursors for these modifications while simultaneously affecting the activity of the enzymes that govern them.

Secondly, nuclear metabolic enzymes, exemplified by but not limited to PKM2, orchestrate gene transcription via distinct pathways, extending beyond their conventional metabolic roles. For instance, PKM2 is capable of phosphorylating histone H3 to directly modify chromatin architecture. Beyond these traditionally understood metabolic functions, metabolic enzymes can engage in direct interactions with epigenetic regulatory proteins (e.g., PFKFB4‐regulated SRC‐3, LDHA‐regulated DOT1L, FBP1‐regulated EZH2, GAPDH‐regulated Sirt1, and HDAC2. Furthermore, metabolic enzymes can enter into direct interactions with transcription factors or coregulators, as evidenced by PKM2‐phosphorylated STAT3 and PKM2‐regulated HIF.

To illuminate the intricate mechanisms governing the translocation of key metabolic enzymes or metabolites to the nuclear compartment, and to understand their complex functionalities therein, is to open new vistas in cancer therapeutics. Given the pleiotropic roles of these entities, targeted inhibition could offer a particularly efficacious approach in cancer treatment. These strategies encompass: (1) targeting nuclear metabolic enzymes; (2) modulating nuclear‐specific modifications; (3) interfering with the trafficking of metabolites or metabolic enzymes; and (4) targeting modulators of these metabolic enzymes. However, the ultimate efficacy of these novel therapeutic modalities is contingent upon an enhanced understanding of the specific metabolic requirements of tumor cells and the concomitant development of innovative antimetabolic strategies.

The metabolic underpinnings of cancer are likely more expansive than currently anticipated, potentially influencing every facet of neoplastic progression. Advancements in research focusing on nuclear metabolic signaling—still in its nascent stage—will undoubtedly yield groundbreaking insights and perhaps paradigm shifts in our understanding of tumorigenesis. The identification of novel metabolic enzymes within the nuclear compartment, accompanied by a comprehensive elucidation of their functional roles and regulatory mechanisms, will enrich our understanding of tumor metabolism and highlight hitherto undiscovered targets for future pharmacological interventions.

## AUTHOR CONTRIBUTIONS

P. Y. and C. C. conceived the manuscript; Y. C., J. X., and X. L. wrote the initial draft; L. G., J. X., C. C., and Y. C. prepared the figure; C. C. revised the manuscript. All authors have read and approved the final manuscript.

## CONFLICT OF INTEREST STATEMENT

The authors declare no conflict of interest.

## ETHICS STATEMENT

Not applicable.

## Data Availability

Not applicable.
